# Ponatinib after failure of second‐generation tyrosine kinase inhibitor in resistant chronic‐phase chronic myeloid leukemia

**DOI:** 10.1002/ajh.26686

**Published:** 2022-08-30

**Authors:** Hagop M. Kantarjian, Elias Jabbour, Michael Deininger, Elisabetta Abruzzese, Jane Apperley, Jorge Cortes, Charles Chuah, Daniel J. DeAngelo, John DiPersio, Andreas Hochhaus, Jeffrey Lipton, Franck E. Nicolini, Javier Pinilla‐Ibarz, Delphine Rea, Gianantonio Rosti, Philippe Rousselot, Neil P. Shah, Moshe Talpaz, Shouryadeep Srivastava, Xiaowei Ren, Michael Mauro

**Affiliations:** ^1^ Department of Leukemia The University of Texas MD Anderson Cancer Center Houston Texas USA; ^2^ Division of Hematology and Oncology, Department of Medicine University of Utah Huntsman Cancer Institute Salt Lake City Utah USA; ^3^ Division of Hematology, S. Eugenio Hospital Tor Vergata University Rome Italy; ^4^ Centre for Haematology Imperial College London London UK; ^5^ Georgia Cancer Center Augusta Georgia USA; ^6^ Department of Haematology Singapore General Hospital, Duke‐NUS Medical School Singapore Singapore; ^7^ Department of Medical Oncology Dana‐Farber Cancer Institute Boston Massachusetts USA; ^8^ Division of Oncology Washington University School of Medicine St. Louis Missouri USA; ^9^ Department of Hematology/Oncology Universitätsklinikum Jena Jena Germany; ^10^ Princess Margaret Cancer Centre Toronto Ontario Canada; ^11^ Centre Leon Berard, Department d'Hématologie & INSERM U1052 Equipe BMP, Niche Tumorale et Resistance, CRCL Lyon France; ^12^ H. Lee Moffitt Cancer Center & Research Institute Tampa Florida USA; ^13^ Department of Hematology Hopital Saint‐Louis Paris France; ^14^ IRST/IRCCS “Dino Amadori” Meldola Province of Forlì‐Cesena Italy; ^15^ Hospital Mignot University de Versailles Saint‐Quentin‐en‐Yvelines Paris France; ^16^ Department of Medicine (Hematology/Oncology) University of California San Francisco San Francisco California USA; ^17^ Comprehensive Cancer Center University of Michigan Ann Arbor Michigan USA; ^18^ Takeda Development Center Americas, Inc. Lexington Massachusetts USA; ^19^ Memorial Sloan Kettering Cancer Center New York New York USA

## Abstract

Ponatinib, the only third‐generation pan‐BCR::ABL1 inhibitor with activity against all known *BCR::ABL1* mutations including T315I, has demonstrated deep and durable responses in patients with chronic‐phase chronic myeloid leukemia (CP‐CML) resistant to prior second‐generation (2G) TKI treatment. We present efficacy and safety outcomes from the Ponatinib Philadelphia chromosome–positive acute lymphoblastic leukemia (Ph+ ALL) and CML Evaluation (PACE) and Optimizing Ponatinib Treatment in CP‐CML (OPTIC) trials for this patient population. PACE (NCT01207440) evaluated ponatinib 45 mg/day in CML patients with resistance to prior TKI or T315I. In OPTIC (NCT02467270), patients with CP‐CML and resistance to ≥2 prior TKIs or T315I receiving 45 or 30 mg/day reduced their doses to 15 mg/day upon achieving ≤1% *BCR::ABL1* ^IS^ or received 15 mg/day continuously. Efficacy and safety outcomes from patients with CP‐CML treated with ≥1 2G TKI (PACE, *n* = 257) and OPTIC (*n* = 93), 45‐mg starting dose cohort, were analyzed for *BCR::ABL1* ^IS^ response rates, overall survival (OS), progression‐free survival (PFS), and safety. By 24 months, the percentages of patients with ≤1% *BCR::ABL1* ^IS^ response, PFS, and OS were 46%, 68%, and 85%, respectively, in PACE and 57%, 80%, and 91%, respectively, in OPTIC. Serious treatment‐emergent adverse events and serious treatment‐emergent arterial occlusive event rates were 63% and 18% in PACE and 34% and 4% in OPTIC. Ponatinib shows high response rates and robust survival outcomes in patients whose disease failed prior to 2G TKIs, including patients with T315I mutation. The response‐based dosing in OPTIC led to improved safety and similar efficacy outcomes compared with PACE.

## Graphical abstract

Analysis of the largest CP‐CML population in a post‐second‐generation TKI setting (*N* = 350)

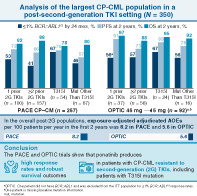

## 1 BACKGROUND

Chronic myeloid leukemia (CML) is a myeloproliferative disorder caused by the constitutively active tyrosine kinase, BCR::ABL1, generated as the result of a reciprocal translocation between chromosomes 9 and 22.^1^ Although tyrosine kinase inhibitors (TKIs) targeting *BCR::ABL1* mutations have improved the therapeutic outcomes in patients with CML,^1^, ^2^ resistance to BCR::ABL1 TKI treatment can occur.^3^ Specifically, the “gatekeeper” mutation in *BCR::ABL1*, T315I, confers a high degree of resistance to multiple TKIs.^4^ Patients with chronic‐phase chronic myeloid leukemia (CP‐CML) resistant to a second‐generation (2G) BCR::ABL1 TKI experience low response rates and poor long‐term outcomes when treated with another 2G TKI.^5^, ^6^, ^7^

Ponatinib is a third‐generation TKI designed to inhibit BCR::ABL1, including the T315I mutation.^8^ The efficacy and safety of ponatinib have been evaluated in two phases 2 trials: Ponatinib Philadelphia chromosome–positive acute lymphoblastic leukemia (Ph+ ALL) and CML Evaluation (PACE, NCT01207440) and Optimizing Ponatinib Treatment in CP‐CML (OPTIC, NCT02467270).^9^, ^10^ PACE was a pivotal phase 2 trial that evaluated the safety and efficacy of ponatinib at an initial dose of 45 mg in patients with heavily pretreated CML or Ph+ ALL with resistance or unacceptable side effects to 2G TKI treatment or with the *BCR::ABL1* T315I mutation.^9^, ^11^ In PACE, patients had deep, lasting responses to ponatinib regardless of baseline *BCR::ABL1* mutation status.^9^, ^11^

OPTIC is an ongoing prospective phase 2 trial of a response‐based dose‐reduction strategy to optimize efficacy while attempting to minimize cardiovascular (CV) risks of ponatinib among patients who had resistance to ≥2 prior TKIs or with the *BCR::ABL1* T315I mutation.^10^ In OPTIC, patients were randomly assigned to starting doses of ponatinib of 45, 30, or 15 mg. Responders, defined as patients with *BCR::ABL1* ^*IS*^ transcript levels of ≤1%, in the 45‐ and 30‐mg groups had their dose reduced to 15 mg.^10^ The results from OPTIC demonstrated clinical benefit and a manageable safety profile with response‐based ponatinib dosing.^10^ This is the largest analysis of post‐2G TKI patient populations, including patients with the T315I mutation, in CML, performed to date.

## 2 METHODS

### 2.1 Study design and treatment

PACE was a phase 2, single‐arm study in which patients received an initial oral dose of ponatinib 45 mg once daily.^9^ Treatment then continued until there was evidence of disease progression, an adverse event (AE) occurred that required discontinuation, the patient withdrew, or the investigator elected treatment termination.^9^ Dose reductions from 45 to 30 mg/day or 15 mg/day once daily were enacted to manage AEs and toxicity, per protocol and FDA mandate, or implemented proactively because of concerns regarding the risk of AOEs which were observed with continued follow up.^11^

OPTIC is an ongoing multicenter, phase 2 trial in which patients were randomly assigned (1:1:1 ratio) to an initial oral dose of ponatinib 45, 30, or 15 mg once daily.^10^ Patients who received ponatinib 45 and 30 mg reduced their dose to 15 mg at 3, 6, 9, or 12 months upon achievement of response, which was defined as ≤1% *BCR::ABL1* transcript level on the International Scale (*BCR::ABL1* ^*IS*^).^10^

For both studies, the study protocols, amendments, and informed consent form were approved by the institutional review board/ethics committee of each participating study center.^9^, ^10^

### 2.2 Patient population

Complete inclusion and exclusion criteria have been published with the primary analyses.^9^, ^10^ All patients in PACE and OPTIC provided written informed consent.^9^, ^10^

Patients enrolled in PACE were adults (≥18 years of age) with resistant/intolerant CML or Ph+ ALL resistant to the 2G TKIs dasatinib or nilotinib or with the *BCR::ABL1* T315I mutation.^9^

Patients enrolled in OPTIC were adults (≥18 years of age) with CP‐CML resistant to ≥2 prior TKIs or with a *BCR::ABL1* T315I mutation.^10^

### 2.3 Efficacy assessments

Response assessments included cytogenetic responses by bone marrow assessment at month 12 (partial cytogenetic response [PCyR], complete cytogenetic response [CCyR] or major cytogenetic response [MCYR]) and hematologic response by peripheral blood samples at 3‐month intervals.

To investigate ponatinib efficacy among patients with CP‐CML resistant to prior BCR::ABL1 2G TKI, the response outcomes ≤10%, ≤1%, ≤0.1%, ≤0.01%, ≤0.0032% *BCR::ABL1* ^*IS*^, PFS, and OS were evaluated. The molecular response was assessed by *BCR::ABL1* ^*IS*^ measurement via quantitative real‐time polymerase chain reaction (PCR) of the *BCR::ABL1* transcript at a central molecular diagnostics laboratory (MolecularMD); results were reported to the participating investigator.^9^, ^10^

Progression‐free survival (PFS) was defined as the interval between the first dose of ponatinib and disease progression (progression to accelerated‐phase [AP]‐CML or blast‐phase [BP]‐CML), loss of complete hematologic response (CHR) or MCyR, or doubling of white blood cell count to >20 000/mm^3^ on 2 occasions ≥4 weeks apart in patients without CHR or death from any cause.^9^ Overall survival (OS) was defined as the interval between the first dose of ponatinib and death from any cause.^9^ Response and survival criteria are outlined in Table S1.

### 2.4 Safety assessments

AEs were continuously assessed throughout each study and were graded according to the National Cancer Institute Common Terminology Criteria for Ad, version 4.0.^9^, ^10^ An independent CV endpoint adjudication committee reviewed all documentation related to arterial occlusive events (AOEs). Complete details of the adjudication committee have been published.^10^

### 2.5 Statistical analysis

Efficacy and safety analyses were conducted in a subset of 257 patients from the CP‐CML cohort of PACE and a subset of 93 patients from OPTIC who received the starting dose of ponatinib 45 mg/day; both subsets had exposure to ≥1 2G TKIs (dasatinib, nilotinib, or bosutinib). The efficacy and safety data were analyzed separately for each study population. Efficacy data through 12, 24, and 60 months are presented for PACE and through 12 and 24 months for OPTIC. Categorical data were summarized by the number and percentage of patients. All data were summarized descriptively.

Exposure‐adjusted AOE rates were calculated as (number of first events in interval)/(total exposure for interval in patient‐years) × 100.

## 3 RESULTS

The data cutoff dates were February 6, 2017, for PACE and May 31, 2020, for OPTIC. For this analysis, 257 patients from the PACE CP‐CML cohort and 93 from the OPTIC 45 mg starting dose cohort were analyzed. The median follow‐up time for patients in PACE was 57 months (0.1–73); the median follow‐up time in OPTIC was 32 months (1–57).^10^, ^11^ The median dose intensity at 24 months for the post 2G population was 30 mg for PACE and 15 mg for OPTIC.

### 3.1 Patient disposition and baseline characteristics

In total, this analysis included patients with CP‐CML who had ≥1 prior 2G TKI and received a starting dose of ponatinib 45 mg in both trials (Table S2). All patients were resistant to ≥1 prior 2G TKI, with most patients (98% in PACE and 100% in OPTIC) having received ≥2 prior 2G TKIs; 62% of patients in PACE and 54% of patients in OPTIC received ≥3 prior 2G TKIs. At baseline, only 5% of patients in PACE and 3% in OPTIC had achieved ≤1% *BCR::ABL* ^*IS*^, and 27% of patients in PACE and 39% in OPTIC had complete hematologic response as their best response to last prior TKI. Almost half of the patients, 47% in PACE and 43% in OPTIC, had any *BCR::ABL1* secondary kinase domain mutation at baseline, including 21% and 26% of patients with the T315I mutation in PACE and in OPTIC, respectively. Over half of patients, 53% in PACE and 56% in OPTIC, had no *BCR::ABL1* mutation at baseline. One patient in OPTIC had an unknown mutation status. Most patients stopped prior therapy due to resistance: 96% (*n* = 247) in PACE and 98% (*n* = 91) in OPTIC.

### 3.2 Efficacy

**FIGURE 1:**
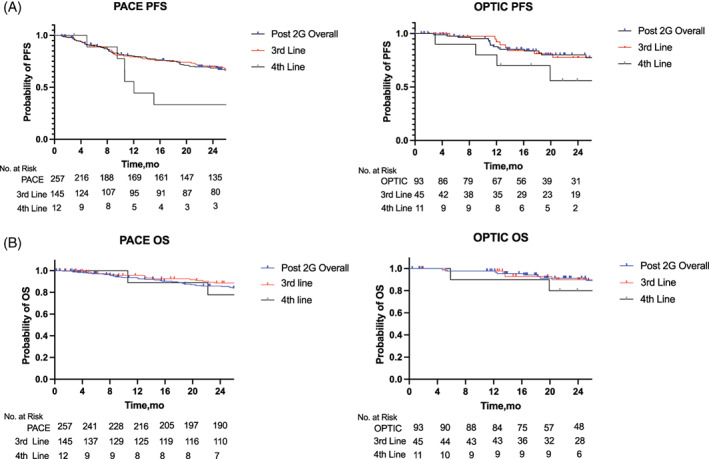
Progression‐free survival and overall Survival in PACE CP‐CML and OPTIC 45 mg→15 mg post‐2G TKI populations. Kaplan–Meier survival curves depicting (A) PACE and OPTIC PFS (B) PACE and OPTIC OS. Blue curves represent patients in the PACE trial and red curves represent patients in the OPTIC trial. Number at risk is indicated at the bottom of the graph for each time point. CP‐CML, chronic‐phase chronic myeloid leukemia; OPTIC, optimizing ponatinib treatment in CP‐CML; OS, overall survival; PACE, positive acute lymphoblastic leukemia (Ph+ ALL) and CML Evaluation; PFS, progression‐free survival; TKI, tyrosine kinase inhibitor

In PACE, 42%, 46%, and 47% of patients achieved ≤1% *BCR::ABL1* ^*IS*^ response by 12, 24, and 60 months, respectively (Table 1). In OPTIC, 52% and 57% achieved ≤1% *BCR::ABL1* ^*IS*^ response by 12 and 24 months, respectively. For survival outcomes, in PACE, 68% of patients reached 2‐year PFS and 85% of patients reached 2‐year OS, and in OPTIC, 80% of patients reached 2‐year PFS and 91% of patients reached 2‐year OS (Table 1; Figure 1). When broken down by line of therapy, OS remained similar for patients treated with ponatinib in the third and fourth line in both PACE and OPTIC; however, PFS dropped in the fourth line in both PACE and OPTIC.

**TABLE 1:** Ponatinib efficacy by number of prior second‐generation TKIs

Response	PACE CP‐CML (*n* = 257)			OPTIC 45 mg→15 mg (*n* = 93)		
	1 prior 2G TKI (*n* = 100)	≥2 prior 2G TKIs (*n* = 157)	Overall (*n* = 257)	1 prior 2G TKI (*n* = 37)	≥2 prior 2G TKIs (*n* = 56)	Overall (*n* = 92)
≤1% *BCR::ABL1* ^*IS*^ by:						
12 months, %	47	39	42	47^a^	55	52^a^
24 months, %	53	41	46	56^a^	57	57^a^
60 months, %	56	41	47	NA	NA	NA
≤0.1% *BCR::ABL1* ^*IS*^ by:						
12 months, %	32	26	28	19	20	20
24 months, %	38	31	34	39	30	34
60 monthss, %	41	34	37	NA	NA	NA
PFS at:						
2 years, %	72	66	68	91	73	80
5 years, %	66	49	55	NA	NA	NA
OS at:						
2 years, %	82	88	85	97	88	91
5 years, %	77	73	75	NA	NA	NA

Abbreviations: CP‐CML, chronic‐phase chronic myeloid leukemia; OPTIC, optimizing ponatinib treatment in CP‐CML; OS, overall survivall; PACE, positive acute lymphoblastic leukemia (Ph+ ALL) and CML Evaluation; PFS, progression‐free survival; TKI, tyrosine kinase inhibitor.

### 3.3 Efficacy by number of prior second‐generation TKIs

Of the patients exposed to ≥2 prior 2G TKIs in PACE, 39% and 41% achieved ≤1% *BCR::ABL1* ^*IS*^ responses through 12 and 24 months, respectively, compared with 47% and 53% for patients exposed to 1 prior 2G TKI. In a similar patient population with exposure to ≥2 prior 2G TKIs in OPTIC, 55% and 57% achieved ≤1% *BCR::ABL1* ^*IS*^ responses through 12 and 24 months, respectively, compared with 47% and 56% for patients exposed to 1 prior 2G TKI (Table 1). In PACE, rates of 2‐year PFS and OS were 66% and 88%, respectively, among patients with ≥2 prior 2G TKIs, compared with 72% and 82% among patients with 1 prior 2G TKI. In OPTIC, rates of 2‐year PFS and OS were 73% and 88%, respectively, among patients with ≥2 prior 2G TKIs compared with 91% and 97%, respectively, among patients with 1 prior 2G TKI.

### 3.4 Efficacy by *BCR::ABL1* mutation status

In both trials, ≤1% *BCR::ABL1* ^*IS*^ responses by 12‐ and 24‐month were generally higher for patients with the T315I mutation (PACE, 59% and 67%; OPTIC, 67% and 67%) compared with those with no *BCR::ABL1* mutation (PACE, 38% and 41%; OPTIC, 46% and 54%) and those with a mutation other than T315I (PACE, 43% and 46%; OPTIC, 56% and 56%) (Table 2). Two‐year PFS was lower among patients in PACE with a *BCR::ABL1* mutation other than T315I than those without a mutation or with the T315I mutation (57% vs. 71%, 70%, respectively). In contrast, 2‐year PFS was similar regardless of mutational status among patients in OPTIC: 78% for patients with a mutation other than T315I, 78% for patients without a *BCR::ABL1* mutation, and 85% for patients with the T315I mutation. The 2‐year OS was high among patients with no mutations (PACE, 91%; OPTIC, 89%) and among patients with any mutation (PACE, 79%; OPTIC, 94%). Additional efficacy outcomes by mutation status and prior 2G TKI are provided in Tables S3 and S4. Among patients with the T315I mutation, 70% in PACE (38/54) and 83% in OPTIC (20/24) had their dose reduced; however, 32% of these patients in PACE and 55% of these patients in OPTIC had their dose re‐escalated (Table S5).

**TABLE 2:** Ponatinib efficacy by baseline mutation status

Response	PACE CP‐CML post–2G TKI (*n* = 257)				OPTIC 45 mg→15 mg post–2G TKI (*n* = 92)^a^ ^,^ ^b^			
	Mutation status				Mutation status			
	None (*n* = 136)	T315I (*n* = 54)	Mutation other than T315I (*n* = 67)	Any (*n* = 121)	None (*n* = 52)	T315I (*n* = 24)	Mutation other than T315I (*n* = 16)	Any (*n* = 40)
≤1% *BCR::ABL1* ^*IS*^ by^c^:								
12 months, %	38	59	43	50	46	67	56	63
24 months, %	41	67	46	55	54	67	56	63
60 months, %	43	69	46	56	54	67	56	63
PFS at:								
24 months, %	71	70	57	63	78	85	78	82
60 months, %	58	47	46	45	NA	NA	NA	NA
OS at:								
24 months, %	91	78	80	79	89	95	92	94
60 months, %	80	62	67	64	NA	NA	NA	NA

Abbreviations: CP‐CML, chronic‐phase chronic myeloid leukemia; NA, not applicable; OPTIC, optimizing ponatinib treatment in CP‐CML; OS, overall survival; PACE, positive acute lymphoblastic leukemia (Ph+ ALL) and CML Evaluation; PFS, progression‐free survival; TKI, tyrosine kinase inhibitor.

^a^ OPTIC: One patient did not have *BCR::ABL1* and was excluded from the ITT population for ≤1% *BCR::ABL1* ^*IS*^ response rates.

^b^ One patient is missing baseline mutation information.

^c^ Patients who had achieved ≤1% *BCR::ABL1* ^*IS*^ at baseline are included in this analysis.

### 3.5 Safety and tolerability

The incidence of treatment‐emergent adverse events (TEAEs) was impacted by the number of prior 2G TKIs (Tables 3 and S6). In PACE, the incidence of treatment‐emergent serious adverse events (TE‐SAE) was lower in patients treated with ≤2 prior 2G TKIs versus ≥3 prior 2G TKIs, while the incidence of treatment‐emergent AOEs (TE‐AOEs) and serious TE‐AOEs were similar regardless of line of therapy. In OPTIC, which employed the response‐based dosing regimen strategy, the overall incidence of AOEs was lower in patients treated with ≤2 prior 2G TKIs versus ≥3 prior 2G TKIs.

**TABLE 3:** Safety by number of prior second‐generation TKIs

Adverse event	PACE CP‐CML (*n* = 257)		OPTIC 45 mg→15 mg (*n* = 93)	
	≤2 prior 2G TKI (*n* = 95)	≥3 prior 2G TKIs (*n* = 162)	≤2 prior 2G TKI (*n* = 43)	≥3 prior 2G TKIs (*n* = 50)
TEAEs %	99	100	100	100
Grade 3–4 TEAEs %	81	81	67	66
TE‐AOEs %	18	16	7	12
Grade 3–4 TE‐AOEs %	12	9	5	6

Abbreviations: AOEs, arterial occlusive events; CP‐CML, chronic‐phase chronic myeloid leukemia; OPTIC, optimizing ponatinib treatment in CP‐CML; PACE, positive acute lymphoblastic leukemia (Ph+ ALL) and CML Evaluation; TEAEs, treatment‐emergent adverse events; TE‐AOEs, treatment‐emergent arterial occlusive events; TKI, tyrosine kinase inhibitor.

**FIGURE 2:**
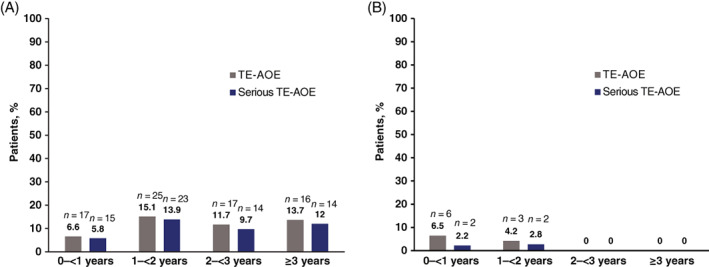
Exposure‐adjusted cumulative treatment‐emergent arterial occlusive events over time. Bar graphs (A) PACE and (B) OPTIC showing exposure‐adjusted cumulative TE‐AOEs by year intervals for the post 2G population. Light gray bars represent TE‐AOEs, and blue bars represent serious TE‐AOEs. Median follow‐up for PACE was 57 months and median follow‐up for OPTIC was 32 months. AOE, arterial occlusive event; CP‐CML, chronic‐phase chronic myeloid leukemia; OPTIC, optimizing ponatinib treatment in CP‐CML; PACE, positive acute lymphoblastic leukemia (Ph+ ALL) and CML Evaluation; TE, treatment‐emergent

In the overall post‐2G populations, exposure‐adjusted adjudicated AOEs per 100 patients per year in the first 2 years were 8.2 in PACE and 5.6 in OPTIC. The exposure‐adjusted incidence of TE‐AOEs and serious TE‐AOEs was lower among patients in OPTIC than in PACE for <2 years of treatment (Figure 2).

## 4 DISCUSSION

Our analysis demonstrates that treatment with ponatinib at an initial dose of 45 mg supports high response rates and survival outcomes among patients with CP‐CML resistant to 2G TKIs, regardless of *BCR::ABL1* mutation status. Patients with CP‐CML whose disease becomes resistant to a 2G BCR::ABL1 TKI, either with or without a *BCR::ABL1* kinase domain mutation, experience low response rates, and poor long‐term outcomes if treated with another 2G BCR::ABL1 TKI.^5^, ^6^ For example, 2 studies have shown that the probability of complete cytogenic response and MCyR in patients with CML treated with a 2G TKI who experienced failure of second‐line TKI therapy is 22%–26% and 33%, respectively.^5^, ^7^

OPTIC was designed to evaluate the efficacy and safety of lowering the ponatinib dose once patients achieve treatment response. Data from the current analysis indicate that efficacy was similar, if not better, with response‐based dosing in OPTIC compared with patients with similar demographics in PACE. Sustained responses were observed in both studies in patients with CP‐CML resistant to ≥1 2G TKI regardless of T315I status or number of prior 2G TKIs. Although the definition of an acceptable response is not clearly formalized for third‐line treatment and beyond, achieving ≤1% *BCR::ABL1* ^*IS*^ response is considered an important endpoint with meaningful survival implications, further supporting the robust efficacy results observed with ponatinib treatment in third‐line therapy.^12^

AOEs have been identified as a notable AE for ponatinib in the PACE trial.^11^ Additionally, CV events have been reported among patients with CP‐CML receiving any generation TKI, which can contribute to treatment discontinuation.^13^, ^14^, ^15^, ^16^, ^17^, ^18^ This analysis showed that in both trials, patients treated with ≤2 prior 2G TKIs had fewer TE‐SAEs than patients treated with ≥3 prior 2G TKIs. In OPTIC, which employed the response‐based dosing regimen strategy, patients had a reduced incidence of exposure‐adjusted TE‐AOEs and AOEs when compared with PACE.

Together, these data demonstrate improved and sustained outcomes and highlight the clinical benefit of ponatinib treatment in this difficult‐to‐treat population of patients with CP‐CML who had disease resistant to 2G TKIs, regardless of the line in therapy and mutation status. These results are consistent with previous studies showing that patients with CP‐CML with resistance after second‐line treatment with prior 2G TKIs are approximately twice as likely to achieve CCyR when treated with ponatinib rather than a 2G TKI^7^ and that ponatinib is an optimal TKI in this patient population.^19^

Asciminib, a Specifically Targeting the ABL Myristoyl Pocket (STAMP) inhibitor that blocks the kinase activity of BCR::ABL1, also has potential activity against the T315I mutation. Currently, there are limited data on the use of asciminib in an exclusive post‐2G setting,^20^ and responses in the T315I mutation patient population were seen at doses much higher (5×) than in those without the T315I mutation.^21^ Head‐to‐head, randomized trials of asciminib versus ponatinib would be needed to compare the efficacy and safety of these agents. However, our analysis represents the largest population of patients with CP‐CML studied in the difficult‐to‐treat post‐2G TKI setting. Data from the PACE and OPTIC trials highlight the important clinical benefits obtained with ponatinib in this difficult‐to‐treat population of patients with disease resistance to 2G TKIs.

Limitations to this analysis include that PACE and OPTIC were open‐label trials, the differences in dosing strategy between the 2 trials, and patient baseline characteristics. The present study was an analysis of 2 separate trials, and therefore the results could not be directly compared. Additionally, although the assessment of AOEs was prospective in both studies, the adjudication in PACE was retrospective. These differences are presented only to assess the consistency of the data.

In this analysis, patients with CP‐CML resistant to 2G TKI treatment, including those with a T315I mutation, demonstrated high response rates and substantial survival outcomes when treated with ponatinib. With the response‐based dosing regimen in OPTIC, efficacy was consistent with that of PACE in patients with CP‐CML resistant to 2G TKI therapy. Overall, this analysis demonstrated a favorable benefit to the risk profile for ponatinib in patients who have failed 2G TKI(s), regardless of T315I status. Although the utilization of ponatinib as second‐line therapy for CP‐CML is limited, the efficacy of ponatinib in the post‐2G TKI setting demonstrated in this analysis has the potential to move it into second‐line therapy for more patients with CP‐CML.

## CONFLICT OF INTEREST

Hagop M. Kantarjian: Honoraria (AbbVie, Amgen, ARIAD Pharmaceuticals, Bristol Myers Squibb, ImmunoGen, Orsenix, Pfizer, Agios, Takeda, Actinium Pharmaceuticals); research funding (all to institution: Pfizer, Amgen, Bristol Myers Squibb, Novartis, ARIAD Pharmaceuticals, Astex Pharmaceuticals, AbbVie, Agios, Cyclacel, ImmunoGen, Jazz Pharmaceuticals). Elias Jabbour: Research grants and advisory roles (AbbVie, Adaptive Biotechnologies, Amgen, Bristol Myers Squibb, Genentech, Pfizer, Takeda). Michael Deininger: Consultancy, membership on an entity's board of directors or advisory committees, part of a study management committee and research funding (Blueprint Medicines); consultancy (Fusion Pharma, Medscape, DisperSol); consultancy, membership on an entity's board of directors or advisory committees, and research funding (Takeda); consultancy and membership on an entity's board of directors or advisory committees (Sangamo); consultancy and research funding (Novartis); consultancy, honoraria, and research funding (Incyte); research funding (SPARC, DisperSol, Leukemia & Lymphoma Society). Elisabetta Abruzzese: Advisory board/consultancy (Incyte, Novartis, Pfizer); honoraria (Bristol Myers Squibb). Jane Apperley: Honoraria, research funding and speaker's bureau (Incyte, Pfizer); honoraria and speakers bureau (Bristol Myers Squibb, Novartis). Jorge Cortes: Consultancy and research funding (Bristol Myers Squibb, Daiichi Sankyo, Jazz Pharmaceuticals, Astellas, Novartis, Pfizer, Takeda, BioPath Holdings); research funding (Sun Pharma, Telios Pharma, Arog Pharmaceuticals, Merus, ImmunoGen); membership on an entity's board of directors or advisory committees (BioPath Holdings); consultancy (Amphivena Therapeutics, BioLineRx). Charles Chuah: Honoraria (Novartis, Korea Otsuka Pharmaceutical, Otsuka [Philippines] Pharmaceutical, Steward Cross: Korea Otsuka International Asia Arab); honoraria (Novartis, Bristol Myers Squibb); travel and research funding (Pfizer). Daniel J. DeAngelo: Consulting/advisory role (Amgen, Autolus, Blueprint Medicines, Forty‐Seven, Gilead, Incyte, Jazz, Novartis, Pfizer, Servier, and Takeda), research funding (all to institution: Novartis, AbbVie, GlycoMimetics, Blueprint Medicines). John DiPersio: Equity ownership (Magenta Therapeutics, WUGEN); board/advisory membership (RiverVest Venture Partners); research funding (Amphivena Therapeutics, NeoImmuneTech, MacroGenics, Incyte, BioLineRx, WUGEN); speaking fees (Incyte), patents/pending patents (CART with Wash University and WUGEN, VLA‐4 Inhibitors with Washington University and Magenta Therapeutics). Andreas Hochhaus: Research funding (Bristol Myers Squibb, Novartis, Pfizer, Incyte). Jeffery H. Lipton: Consultancy and research funding (Bristol Myers Squibb, ARIAD Pharmaceuticals, Pfizer, Novartis). Franck E. Nicolini: Honoraria, speakers bureau, travel/accommodations/expenses (Novartis, Incyte, Pfizer); consulting/advisory role (Sun Pharma, Novartis, Analysis group, Kartos Therapeutics); research fundings all to institutions (Incyte Biosciences, Novartis); board entity (Novartis, Pfizer; Incyte). Javier Pinilla‐Ibarz: Consulting/advisory role (AbbVie, Janssen, AstraZeneca, Novartis, TG Therapeutics, Takeda); speakers bureau (AbbVie, Janssen, AstraZeneca, Takeda); research funding (MEI, Sunesis); patents/royalties/other intellectual property (Sellas). Delphine Rea: Steering committee (Novartis); advisory board (Incyte, Pfizer, Novartis). Gianantonio Rosti: Steering Committee and advisory board (Novartis, Pfizer, Incyte); speaker bureau (Novartis, Incyte, BMD, and Pfizer). Philippe Rousselot: Consultancy and research funding (Incyte, Pfizer); consultancy (Bristol Myers Squibb, Novartis, Takeda). Neil Shah: Research funding (Bristol Myers Squibb). Moshe Talpaz: Grant/research support (Takeda, Novartis). Shouryadeep Srivastava: Employment (Takeda). Xiaowei Ren: Employment (Takeda). Michael Mauro: Consultancy, honoraria, travel, accommodation, expenses, and research funding (Bristol Myers Squibb, Novartis, Takeda, Pfizer); research funding (Sun Pharma/SPARC).

## Supporting information


**Table S1** Response and survival criteria.
**Table S2**. Patient baseline characteristics of post‐2G TKI population.
**Table S3**. Ponatinib efficacy; ≤10%, ≤1%, ≤0.1%, ≤0.01%, ≤0.0032% *BCR::ABL1*; by mutation status.
**Table S4**. Ponatinib efficacy; ≤10%, ≤1%, ≤0.1%, ≤0.01%, ≤0.0032% by prior second‐generation TKI.
**Table S5**. Dose reduction and escalation for patients with T315I mutation.
**Table S6.** Most common TEAEs by 24 months.

## Data Availability

The data sets, including the redacted study protocol, redacted statistical analysis plan, and individual participant data supporting the results reported in this article, will be made available within 3 months from initial request to researchers who provide a methodologically sound proposal. The data will be provided after de‐identification, in compliance with applicable privacy laws, data protection, and requirements for consent and anonymization.
