# Supplementary material for: Ponatinib after failure of second‐generation tyrosine kinase inhibitor in resistant chronic‐phase chronic myeloid leukemia
Source: Am J Hematol. 2022 Aug 30;97(11):1419–26. doi: 10.1002/ajh.26686 (PMC9804741; doi:10.1002/ajh.26686)
Supplement: Supplementary file 1 — Table S1 Response and survival criteria. Table S2. Patient baseline characteristics of post‐2G TKI population. Table S3. Ponatinib efficacy; ≤10%, ≤1%, ≤0.1%, ≤0.01%, ≤0.0032% BCR::ABL1; by mutation status. Table S4. Ponatinib efficacy; ≤10%, ≤1%, ≤0.1%, ≤0.01%, ≤0.0032% by prior second‐generation TKI. Table S5. Dose reduction and escalation for patients with T315I mutation. Table S6. Most common TEAEs by 24 months. [file AJH-97-1419-s001.docx]

**Table S1. Response and Survival Criteria**

| **Response** | **OPTIC Criteria** | **PACE Criteria** |
| --- | --- | --- |
| Cytologic response |  |  |
| CCyR | 0% Ph+ metaphases | 0% Ph+ metaphases |
| PCyR | >0%–35% Ph+ metaphases | 1%–35% Ph+ metaphases |
| Molecular response |  |  |
| MR | ≤1% *BCR::ABL1*^IS^ (primary end point) | Molecular Response 4: either detectable transcripts ≤0.01% *BCR::ABL^IS^* or undetectable *BCR::ABL* transcripts in cDNA with ≥10,000 *ABL* transcripts  Molecular Response 4.5: either detectable transcripts ≤0.0032% *BCR::ABL^IS^* or undetectable *BCR::ABL* transcripts in cDNA with ≥32,000 *ABL* transcripts |
| MMR | ≤0.1% *BCR::ABL1*^IS^ | ≤0.1% *BCR::ABL1*^IS^ |
| Hematologic response |  |  |
| CHR | - WBC count ≤ institutional ULN - Platelets <450,000/mm^3^ - No peripheral blood blasts or promyelocytes - <5% myelocytes plus metamyelocytes - Basophils <5% - No extramedullary involvement | - WBC count ≤ institutional ULN - Platelets <450,000/mm^3^ - No peripheral blood blasts or promyelocytes - <5% myelocytes plus metamyelocytes - Basophils <5% - No extramedullary involvement |
| Progression-free survival | - Death - Development of AP- or BP-CML - Loss of CHR (in the absence of CyR) confirmed by development in CBC at least 4 weeks apart - Loss of MCyR by bone marrow cytogenetic assessment - Increasing WBC count in patient without CHR defined by doubling of WBC count to greater than 20,000/μL on 2 occasions at least 4 weeks apart (after the first 4 weeks of therapy) | - Death - Development of AP- or BP-CML - Loss of CHR (in the absence of CyR) confirmed by development in CBC at least 4 weeks apart - Loss of MCyR - Increasing WBC count in patient without CHR defined as doubling of WBC count to greater than 20,000/μL on 2 occasions at least 4 weeks apart (after the first 4 weeks of therapy) |

AP-CML, accelerated-phase chronic myeloid leukemia; ANC, absolute neutrophil count; BP-CML, blast-phase chronic myeloid leukemia; CBC, complete blood count; CCyR, complete cytologic response; CHR, complete hematologic response; CyR, cytologic response; MCyR, major cytologic response; MR, molecular response; MMR, major molecular response; PCyR, partial cytologic response; ULN, upper limit of normal; WBC, white blood cell

## Table S2: Patient Baseline Characteristics of Post-2G TKI Population

| **Characteristic** | **PACE  CP-CML**  **(n=257)** | **OPTIC  45 mg🡲15 mg**  **(n=93)** |
| --- | --- | --- |
| Median age, yrs (range) | 61 (21–94) | 46 (19–81) |
| Male, n (%) | 134 (52) | 49 (53) |
| Median time since Dx, yr (range) | 7 (0.5–27) | 6 (0.7–21) |
| *BCR::ABL1* mutation at baseline, n (%) |  |  |
| No mutation | 136 (53) | 52 (56) |
| Any mutation | 121 (47) | 40 (43)^a^ |
| T315I | 54 (21) | 24 (26) |
| Mutation other than T315I | 203 (79) | 69 (74) |
| CV risk factors, n (%) |  |  |
| Arterial hypertension | 99 (39) | 29 (31) |
| Diabetes mellitus | 33 (13) | 5 (5) |
| Hypercholesterolemia | 65 (25) | 3 (3) |
| Prior therapy stopped for resistance, n (%)^b^ | 247 (96) | 91 (98) |
| Number of prior 2G TKIs, n (%) |  |  |
| 1 | 6 (2) | 0 |
| 2 | 92 (36) | 43 (46) |
| 3 | 146 (57) | 42 (45) |
| 4 | 13 (5) | 8 (9) |
| Best response to prior TKI, n (%)^c^ |  |  |
| None/PD | 61 (24) | 9 (10) |
| CHR | 68 (27) | 36 (39) |
| MCyR | 63 (25) | 27 (46) |
| CCyR | 36 (14) | 16 (17) |
| ≤10% *BCR::ABL1^IS^* | 0 | 1 (1) |
| ≤1% *BCR::ABL1^IS^* | 0 | 0 |
| ≤0.1% *BCR::ABL1^IS^* | 10 (4) | 11 (12) |
| *BCR::ABL1^IS^* at baseline, n (%) |  |  |
| >10% | 194 (76) | 73 (79) |
| >1–10% | 49 (19) | 16 (17) |
| ≤1% | 12 (5) | 3 (3) |

^a^ One patient had unknown mutation status

^b^ 9 patients in PACE and 1 patient in OPTIC stopped prior therapy for intolerance; 1 patient in PACE and 1 patient in OPTIC stopped prior therapy for unspecified “other” reasons

^c^ Best response to last prior TKI was lost (ie, resistant disease) by the time of enrollment in PACE or OPTIC

2G, second generation; CCyR, complete cytogenetic response; CHR, complete hematologic response; CP-CML, chronic-phase chronic myeloid leukemia; CV, cardiovascular; D/C, discontinuation; Dx, diagnosis; MCyR, major cytogenetic response; pts, patients; PD, progressive disease; TKI, tyrosine kinase inhibitor

**Table S3. Ponatinib Efficacy****; ≤10%, ≤1%, ≤0.1%, ≤0.01%, ≤0.0032% *BCR::ABL1*; By Mutation Status**

| **Response, n (%)** | **PACE CP-CML Post–2G TKI**  **(n=257)**  **Mutation Status** | | | | **OPTIC 45 mg** **🡲** **15 mg  Post–2G TKI (n=92)^a,b^**  **Mutation Status** | | | |
| --- | --- | --- | --- | --- | --- | --- | --- | --- |
|  | **None  (n=136)** | **T315I (n=54)** | **Mutation other than T315I (n=67)** | **Any (n=121)** | **None (n=52)** | **T315I (n=24)** | **Mutation other than T315I (n=16)** | **Any (n=40)** |
| ≤10% *BCR::ABL1:* |  |  |  |  |  |  |  |  |
| 12 months | 79 (58.1) | 36 (66.7) | 43 (64.2) | 79 (65.3) | 37 (71.2) | 18 (75.0) | 10 (62.5) | 28 (70.0) |
| 24 months | 85 (62.5) | 39 (72.2) | 45 (67.2) | 84 (69.4) | 37 (71.2) | 18 (75.0) | 10 (62.5) | 28 (70.0) |
| 60 months | 89 (65.4) | 39 (72.2) | 45 (67.2) | 84 (69.4) | 37 (71.2) | 18 (75.0) | 10 (62.5) | 28 (70.0) |
| ≤1% *BCR::ABL1*^c^: |  |  |  |  |  |  |  |  |
| 12 months | 51 (37.5) | 32 (59.3) | 29 (43.3) | 61 (50.4) | 24 (46.2) | 16 (66.7) | 9 (56.2) | 25 (62.5) |
| 24 months | 56 (41.2) | 36 (66.7) | 31 (46.3) | 67 (55.4) | 28 (53.8) | 16 (66.7) | 9 (56.2) | 25 (62.5) |
| 60 months | 59 (43.4) | 37 (68.5) | 31 (46.3) | 68 (56.2) | 28 (53.8) | 16 (66.7) | 9 (56.2) | 25 (62.5) |
| ≤0.1% *BCR::ABL1:* |  |  |  |  |  |  |  |  |
| 12 months | 25 (18.4) | 28 (51.9) | 22 (32.8) | 50 (41.3) | 6 (11.5) | 7 (29.2) | 6 (37.5) | 13 (32.5) |
| 24 months | 35 (25.7) | 32 (59.3) | 25 (37.3) | 57 (47.1) | 14 (26.9) | 11 (45.8) | 7 (43.8) | 18 (45.0) |
| 60 months | 43 (31.6) | 32 (59.3) | 25 (37.3) | 57 (47.1) | 15 (28.8) | 11 (45.8) | 7 (43.8) | 18 (45.0) |
| ≤0.01% *BCR::ABL1:* |  |  |  |  |  |  |  |  |
| 12 months | 11 (8.1) | 17 (31.5) | 16 (23.9) | 33 (27.3) | 0 | 4 (16.7) | 3 (18.8) | 7 (17.5) |
| 24 months | 21 (15.4) | 24 (44.4) | 18 (26.9) | 42 (34.7) | 1 (1.9) | 5 (20.8) | 3 (18.8) | 8 (20.0) |
| 60 months | 28 (20.6) | 26 (48.1) | 20 (29.9) | 46 (38.0) | 3 (5.8) | 5 (20.8) | 4 (25.0) | 9 (22.5) |
| ≤0.0032% *BCR::ABL1*: |  |  |  |  |  |  |  |  |
| 12 months | 5 (3.7) | 11 (20.4) | 11 (16.4) | 22 (18.2) | 0 | 3 (12.5) | 1 (6.2) | 4 (10.0) |
| 24 months | 14 (10.3) | 19 (35.2) | 15 (22.4) | 34 (28.1) | 0 | 3 (12.5) | 2 (12.5) | 5 (12.5) |
| 60 months | 24 (17.6) | 24 (44.4) | 18 (26.9) | 42 (34.7) | 0 | 4 (16.7) | 3 (18.8) | 7 (17.5) |

^a^ OPTIC: One patient did not have *BCR::ABL1* and was excluded from the ITT population for ≤1% *BCR::ABL1^IS^* response rates

^b^ One patient is missing baseline mutation information

^c^ Patients who had achieved MR2 at baseline are included in this analysis

**Table S4. Ponatinib Efficacy; ≤10%, ≤1%, ≤0.1%, ≤0.01%, ≤0.0032% By Prior Second-Generation TKI**

| **Response, n (%)** | **PACE CP-CML Post–2G TKI**  **(n=257)** | | | **OPTIC 45 mg 🡲 15 mg  Post–2G TKI (n=93)^a^** | | | |
| --- | --- | --- | --- | --- | --- | --- | --- |
|  | **1 prior 2G TKI**  **(N=100)** | **≥2 prior 2G TKI**  **(N=157)** | **Overall (N=257)** | | **1 prior 2G TKI**  **(N=37)** | **≥2 prior 2G TKI**  **(N=56)** | **Overall**  **(N=93)** |
| ≤10% *BCR::ABL1*: |  |  |  | |  |  |  |
| 12 months | 67 (67) | 87 (55) | 154 (60) | | 26 (70) | 38 (68) | 64 (69) |
| 24 months | 72 (72) | 91 (58) | 163 (63) | | 26 (70) | 38 (68) | 64 (69) |
| 60 months | 73 (73) | 94 (60) | 167 (65) | | 26 (70) | 38 (68) | 64 (69) |
| ≤1% *BCR::ABL1*^b^: |  |  |  | |  |  |  |
| 12 months | 47 (47) | 61 (39) | 108 (42) | | 17 (46) | 31 (55) | 48 (52) |
| 24 months | 53 (53) | 64 (41) | 117 (46) | | 20 (54) | 32 (57) | 52 (56) |
| 60 months | 56 (56) | 65 (41) | 121 (47) | | 20 (54) | 32 (57) | 52 (56) |
| ≤0.1% *BCR::ABL1*: |  |  |  | |  |  |  |
| 12 months | 32 (32) | 41 (26) | 73 (28) | | 7 (19) | 11 (20) | 18 (19) |
| 24 months | 38 (38) | 49 (31) | 87 (34) | | 14 (38) | 17 (30) | 31 (33) |
| 60 months | 41 (41) | 54 (34) | 95 (37) | | 15 (41) | 17 (30) | 32 (34) |
| ≤0.01% *BCR::ABL1*: |  |  |  | |  |  |  |
| 12 months | 17 (17) | 27 (17) | 44 (17) | | 3 (8) | 3 (5) | 6 (6) |
| 24 months | 25 (25) | 36 (23) | 61 (24) | | 3 (8) | 5 (9) | 8 (9) |
| 60 months | 31 (31) | 39 (25) | 70 (27) | | 4 (11) | 7 (13) | 11 (12) |
| ≤0.0032% *BCR::ABL1*: |  |  |  | |  |  |  |
| 12 months | 11 (11) | 16 (10) | 27 (11) | | 1 (3) | 3 (5) | 4 (4) |
| 24 months | 19 (19) | 27 (17) | 46 (18) | | 2 (5) | 3 (5) | 5 (5) |
| 60 months | 28 (28) | 35 (22) | 63 (25) | | 2 (5) | 5 (9) | 7 (8) |

^a^ OPTIC: One patient did not have *BCR::ABL1* and was excluded from the ITT population for ≤1% *BCR::ABL1^IS^* response rates

^b^ Patients who had achieved ≤1% *BCR::ABL1* at baseline are not included in this analysis

**Table S5. Dose Reduction and Escalation for Patients with T315I Mutation**

|  | **PACE CP-CML (n=257)** | **OPTIC 45 mg**  **(n=93)** |
| --- | --- | --- |
| T315I, n | 54 | 24 |
| Dose reduced, n/N (%) | 38/54 (70.4) | 20/24 (83.3) |
| Does re-elevated, n/N (%) | 12/38 (31.6) | 11/20 (55.0) |

**Table S6. Most Common TEAEs by 24 Months^a^**

| **Event, n (%)** | **PACE**  **CP-CML**  **(N=257)** | | **OPTIC**  **45 mg 🡲 15 mg**  **(N=93)** | |
| --- | --- | --- | --- | --- |
|  | **Any Grade** | **Grade 3/4** | **Any Grade** | **Grade 3/4** |
| Headache | 106 (41) | 8 (3) | 16 (17) | 0 |
| Dry skin | 103 (40) | 8 (3) | 11 (12) | 0 |
| Thrombocytopenia | 101 (39) | 78 (30) | 40 (43) | 28 (30) |
| Constipation | 99 (39) | 5 (2) | 10 (11) | 0 |
| Abdominal pain | 84 (33) | 18 (7) | 10 (11) | (1) |
| Arthralgia | 79 (31) | 8 (3) | 9 (10) | 0 (0) |
| Fatigue | 76 (30) | 6 (2) | 7 (8) | 1 (1) |
| Hypertension | 72 (28) | 27 (11) | 26 (28) | 8 (9) |
| Nausea | 68 (26) | 4 (2) | 7 (8) | 0 |
| Lipase increased | 65 (25) | 31 (12) | 19 (20) | 10 (11) |
| Pyrexia | 63 (25) | 3 (1) | 15 (16) | 1 (1) |
| Myalgia | 61 (24) | 3 (1) | 7 (8) | 1 (1) |
| Rash erythematous | 55 (21) | 7 (3) | 4 (4) | 0 |
| Pain in extremity | 52 (20) | 6 (2) | 8 (9) | 0 |
| Back pain | 50 (19) | 3 (1) | 7 (8) | 0 |
| Diarrhea | 48 (19) | 3 (1) | 3 (3) | 0 |
| Neutropenia | 46 (18) | 38 (15) | 28 (30) | 17 (18) |
| Abdominal pain upper | 46 (18) | 7 (3) | 9 (10) | 1 (1) |
| Vomiting | 44 (17) | 5 (2) | 4 (4) | 0 |
| Alanine aminotransferase increased | 43 (17) | 12 (5) | 21 (23) | 3 (3) |
| Anaemia | 41 (16) | 22 (9) | 20 (22) | 11 (12) |
| Cough | 40 (16) | 0 | 6 (6) | 0 |
| Asthenia | 42 (16) | 4 (1) | 3 (3) | 1 (1) |
| Dyspnoea | 39 (15) | 8 (3) | 4 (4) | 1 (1) |
| Dizziness | 38 (15) | 1 (0) | 4 (4) | 0 |
| Oedema (peripheral) | 37 (14) | 2 (1) | 2 (2) | 0 |
| Aspartate aminotransferase increased | 37 (14) | 6 (2) | 13 (14) | 0 |
| Bone pain | 34 (13) | 1 (0) | 3 (3) | 0 |
| Muscle spasms | 34 (13) | 0 | 1 (1) | 0 |
| Pruritus | 30 (12) | 0 | 2 (2) | 0 |
| Rash | 29 (11) | 0 | 12 (13) | 0 |
| Erythema | 27 (11) | 3 (1) | 2 (2) | 0 |
| Decreased appetite | 29 (11) | 0 | 0 | 0 |
| Upper respiratory tract infection | 29 (11) | 2 (1) | 7 (8) | 0 |
| Insomnia | 27 (11) | 0 | 5 (5) | 0 |
| Dry mouth | 25 (10) | 0 | 0 | 0 |
| Rash (pruritic) | 25 (10) | 0 | 2 (2) | 0 |
| Nasopharyngitis | 26 (10) | 0 | 4 (4) | 1 (1) |
| Hypertriglyceridemia | 3 (1) | 0 | 14 (15) | 1 (1) |

^a^ All-grade events in ≥10% of patients in any treatment arm
